# S-217622, a SARS-CoV-2 main protease inhibitor, decreases viral load and ameliorates COVID-19 severity in hamsters

**DOI:** 10.1126/scitranslmed.abq4064

**Published:** 2022-11-03

**Authors:** Michihito Sasaki, Koshiro Tabata, Mai Kishimoto, Yukari Itakura, Hiroko Kobayashi, Takuma Ariizumi, Kentaro Uemura, Shinsuke Toba, Shinji Kusakabe, Yuki Maruyama, Shun Iida, Noriko Nakajima, Tadaki Suzuki, Shinpei Yoshida, Haruaki Nobori, Takao Sanaki, Teruhisa Kato, Takao Shishido, William W. Hall, Yasuko Orba, Akihiko Sato, Hirofumi Sawa

**Affiliations:** ^1^ Division of Molecular Pathobiology, International Institute for Zoonosis Control, Hokkaido University, Sapporo, 001-220, Japan.; ^2^ Shionogi & Co., Ltd., Osaka 561-0825, Japan.; ^3^ Laboratory of Biomolecular Science, Faculty of Pharmaceutical Science, Hokkaido University, Sapporo, 060-0812, Japan.; ^4^ Department of Pathology, National Institute of Infectious Diseases, Tokyo, 162-8640, Japan.; ^5^ International Collaboration Unit, International Institute for Zoonosis Control, Hokkaido University, Sapporo, 001-0020, Japan.; ^6^ National Virus Reference Laboratory, School of Medicine, University College of Dublin, 4, Ireland.; ^7^ Global Virus Network, Baltimore, MD, 21201, USA.; ^8^ Institute for Vaccine Research and Development (IVReD), Hokkaido University, Sapporo, 001-0021, Japan.; ^9^ One Health Research Center, Hokkaido University, Sapporo, 001-0020, Japan.

## Abstract

In parallel with vaccination, oral antiviral agents are highly anticipated to act as countermeasures for the treatment of the coronavirus disease 2019 (COVID-19) pandemic caused by severe acute respiratory syndrome coronavirus 2 (SARS-CoV-2). Oral antiviral medication demands not only high antiviral activity, but also target specificity, favorable oral bioavailability, and high metabolic stability. Although a large number of compounds have been identified as potential inhibitors of SARS-CoV-2 infection in vitro, few have proven to be effective in vivo. Here, we show that oral administration of S-217622 (ensitrelvir), an inhibitor of SARS-CoV-2 main protease (M^pro^, also known as 3C-like protease), decreases viral load and ameliorates disease severity in SARS-CoV-2-infected hamsters. S-217622 inhibited viral proliferation at low nanomolar to sub-micromolar concentrations in cells. Oral administration of S-217622 demonstrated favorable pharmacokinetic properties and accelerated recovery from acute SARS-CoV-2 infection in hamster recipients. Moreover, S-217622 exerted antiviral activity against SARS-CoV-2 variants of concern (VOCs), including the highly pathogenic Delta variant and the recently emerged Omicron BA.5 and BA.2.75 variants. Overall, our study provides evidence that S-217622, an antiviral agent that is under evaluation in a phase 3 clinical trial (clinical trial registration no. jRCT2031210350), possesses remarkable antiviral potency and efficacy against SARS-CoV-2 and is a prospective oral therapeutic option for COVID-19.

## Teaser

S-217622 (ensitrelvir) decreases SARS-CoV-2 replication and COVID-19 severity in hamsters.

## INTRODUCTION

During the ongoing severe acute respiratory syndrome coronavirus 2 (SARS-CoV-2) pandemic, an increasing number of coronavirus disease 2019 (COVID-19) cases have been reported, and the virus remains a serious public health concern. Vaccines and antiviral agents for COVID-19 have been developed and some have been approved for clinical use (*1*). Oral antiviral medication is highly anticipated to shift the momentum of the pandemic because patients can administer it by themselves and thus benefit from easy access to treatment (*2*). To date, two orally bioavailable antivirals have been validated in clinical trials and have been used for the treatment of COVID-19: molnupiravir and nirmatrelvir. Molnupiravir (MPV, also known as EIDD-2801) is a ribonucleoside prodrug of N-hydroxycytidine (NHC) and targets the viral RNA polymerase of SARS-CoV-2. Nirmatrelvir (also known as PF-07321332) is a selective inhibitor of SARS-CoV-2 main protease (M^pro^). The in vivo efficacy of both antivirals have been experimentally shown in animal models (*3*–*5*).

SARS-CoV-2 possesses two viral proteases: M^pro^, encoded by the *nsp5* gene, and papain-like protease (PL^pro^), encoded by the *nsp3* gene, which cleave nascent viral polyproteins for maturation in host cells (*1*). Because these proteases play essential roles in the intracellular amplification stage of SARS-CoV-2 and lack human homologues, they are ideal targets for specific antivirals (*6*–*8*). S-217622 (ensitrelvir), a small-molecule inhibitor for SARS-CoV-2 M^pro^, has been identified through large-scale screening and structure-based optimization (*9*). S-217622 showed favorable bioavailability and tolerability in healthy adults in a phase 1 trial, and has currently been evaluated in a phase 3 trial (trial registration no. jRCT2031210350) (*10*). To understand how S-217622 acts as an oral therapeutic for COVID-19, the antiviral potential needs to be characterized in-depth. Here we report on the antiviral activity of S-217622 against SARS-CoV-2 variants of concern (VOCs) in cell culture and in hamsters.

## RESULTS

### S-217622 exhibits in vitro antiviral activity against SARS-CoV-2

**Fig. 1.**
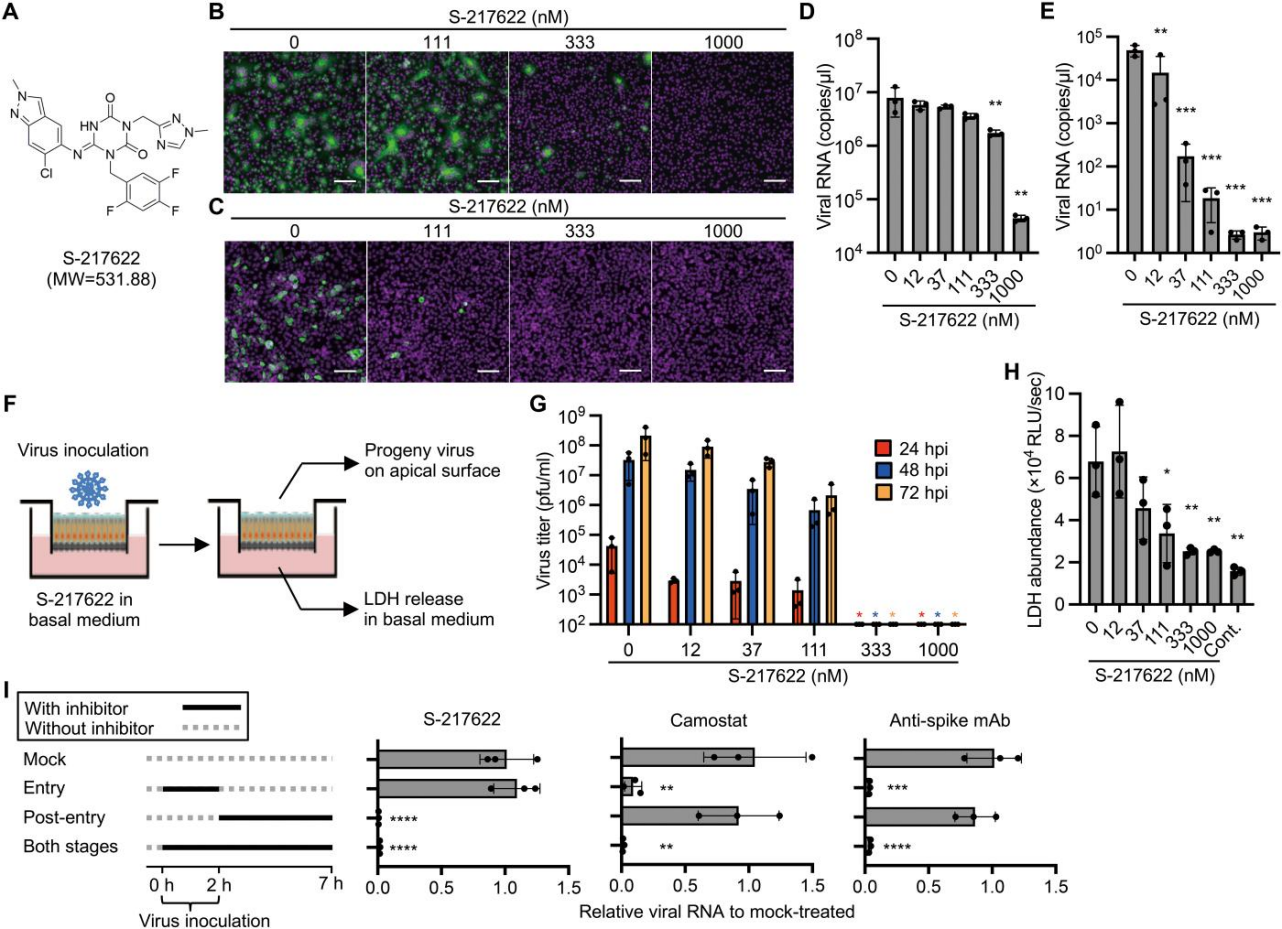
S-217622 inhibits SARS-CoV-2 infection in vitro at the post-entry stage. (**A**)Chemical structure formula of S-217622. (**B** and **C**) Immunofluorescence staining of SARS-CoV-2-infected cells. Vero-TMPRSS2 (**B**) and Calu-3 (**C**) cells were inoculated with a SARS-CoV-2 Delta variant at MOIs of 0.1 or 10, respectively, and then cultured in the presence of S-217622 for 24 hours and 72 hours, respectively. Cells were stained with anti-SARS-CoV-2 nucleocapsid antibody (green) and Hoechst 33342 (magenta). Scale bars, 100 μm. (**D**) Viral RNA concentrations were measured in the culture supernatant of Vero-TMPRSS2 cells at 24 hours post-infection (hpi) with a SARS-CoV-2 Delta variant at an MOI of 0.01. **(E)** Viral RNA concentrations were measured in the culture supernatant of Calu-3 cells at 72 hpi with SARS-CoV-2 Delta variant at an MOI of 0.1. (**F**) Schematic representation of the SARS-CoV-2 infection experiment in a human airway tissue model. Cells were inoculated at the apical surface with 5,000 pfu of SARS-CoV-2. Viral growth was monitored by titration of progeny virus in the mucus layer on apical surface. The tissue damage by viral infection was estimated by measurement of lactate dehydrogenase (LDH) released from cells into the basal culture medium. (**G**) Growth of the SARS-CoV-2 Delta variant in a human airway tissue model. Basal culture medium was supplemented with the indicated concentration of S-217622. (**H**) The abundance of LDH in the basal culture medium was measured by LDH-Glo luminescent assay. (**I**) A time of the addition assay was conducted using S-217622. Calu-3 cells were treated with S-217622 during inoculation (entry stage), after inoculation (post-entry stage), or both entry and post-entry stages of SARS-CoV-2 at an MOI of 3. The inhibitory effect of each treatment was evaluated by measurement of intracellular viral RNA concentrations at 7 hpi. Camostat and anti-SARS-CoV-2 spike protein antibody were used as entry inhibitors for assay controls. The values shown are mean ± standard deviation (SD) of triplicate samples. *p < 0.05, **p < 0.01, ***p < 0.001, ****p < 0.0001 by one-way ANOVA with Dunnett’s test (D, E, H, I) or Kruskal-Wallis test with Dunn’s test (G).

To investigate the antiviral activity of S-217622 (Fig. 1A) in cells, we performed cell-based infection assays using the SARS-CoV-2 Delta variant (lineage B.1.617.2). Infection with SARS-CoV-2 was inhibited in Vero-Transmembrane Serine Protease 2 (TMPRSS2) and Calu-3 cells by S-217622 in a dose-dependent manner (Fig. 1B and C). S-217622 treatment also reduced the progeny viral RNA in the culture supernatants from both Vero-TMPRSS2 and Calu-3 cells in a dose-dependent manner (Fig. 1D and E). No cytotoxicity was observed in the cells at the examined concentrations of S-217622 (fig. S1).

Primary human bronchial epithelial cells (HBE cells) can be differentiated under air-liquid interphase culture conditions and used as an ex vivo model for SARS-CoV-2 infection (*11*). HBE cells were infected with SARS-CoV-2 and treated with S-217622 though the basal medium (Fig. 1F). The progeny virus titers were decreased at low concentrations (12, 37, and 111 nM) and were under the detection limit of the plaque assay, with fewer than 100 plaque forming unit (pfu) per ml, at 333 nM or more of S-217622 (Fig. 1G). Lactate dehydrogenase (LDH) release into basal culture medium is an indicator of cytotoxicity by virus infection and was decreased in the presence of S-217622 (Fig. 1H). The antiviral potency of S-217622 was similar to or higher than those of the antivirals approved for clinical use (nirmatrelvir, molnupiravir, and remdesivir; Table 1). Since nirmatrelvir and remdesivir are major substrates for the plasma membrane multidrug transporter P-glycoprotein (P-gp), these antivirals require a P-gp inhibitor such as CP-100356 to inhibit the efflux of the antivirals from Vero cells, which express high concentrations of P-gp (*12*). CP-100356 enhanced the antiviral activity of S-217622 as well as nirmatrelvir in Vero-TMPRSS2 cells (fig. S2), indicating that S-217622 is a substrate of P-gp. These data indicate that S-217622 inhibits SARS-CoV-2 infection at nanomolar to submicromolar concentrations in different cell types.

**Table 1.** A comparison of the potency of SARS-CoV-2 antivirals by a CPE-based cell viability assay.

	EC_50_ against SARS-CoV-2 Delta		
	293 T-hACE2-TMPRSS2	Vero-TMPRSS2	Vero-TMPRSS2 with CP-100356
**S-217622 (nM)**	26 ± 6.65	407 ± 21.3	69 ± 11.2
**Nirmatrelvir (nM)**	37 ± 5.30	>1000	132 ± 32.0
**NHC (nM)**	538 ± 142	360 ± 49.4	677 ± 8.89
**Molnupiravir (nM)**	3675 ± 1093	3169 ± 217	2280 ± 118
**Remdesivir (nM)**	<3.9	>500	32 ± 4.52

To date, seven variants, namely, Alpha, Beta, Gamma, Delta, Omicron, Lambda, and Mu have emerged and have been assigned as a VOC or variant of interest (VOI) by the WHO. These variants possess amino acid changes in the spike protein, altering the infectivity, transmissibility, pathogenicity, or susceptibility to antibody neutralization (*13*–*16*). Unlike the *S* gene, which encodes the viral spike protein, the *nsp5* gene, which encodes M^pro^, a target for S-217622, is well conserved among these VOCs and VOIs (fig. S3). Indeed, S-217622 exhibited antiviral activity against all VOCs, whereas neutralizing antibodies had different reactivities to some VOCs due to mutations in the spike protein (fig. S4) (*14*, *17*). The remarkable antiviral potency of S-217622 was also reproduced in Vero-TMPRSS2 cells infected with the SARS-CoV-2 Omicron variant (lineage BA.1) (fig. S5). Because the Omicron variant has low fusogenicity, fewer immunostained cells were observed following infection with the Omicron variant compared with the Delta variant.

### S-217622 disrupts the post-entry stage of SARS-CoV-2 infection

To validate the antiviral mechanism of S-217622, we employed a luciferase-based biosensor assay for measurement of SARS-CoV-2 M^pro^ activity (*18*). GloSensor-AVLQS contains a M^pro^-cleavage motif and acts as an indicator of the protease activity of M^pro^. GloSensor-RLKGG is a substrate of another SARS-CoV-2 protease, PL^pro^, and was used as a control. S-217622 inhibited the cleavage and activation of GloSensor-AVLQS by M^pro^ but not the cleavage and activation of GloSensor-RLKGG by PL^pro^, indicating the target specificity of S-217622 (fig. S6). It has been reported that progeny SARS-CoV-2 virions are released in the culture supernatant at 8 to 12 hpi (*19*, *20*). The time of addition assay showed that S-217622 has an effect on the post-entry process (2 to 7 hpi) but not the cellular entry process of infection (0 to 2 hpi) (Fig. 1I). These data suggest that S-217622 blocks SARS-CoV-2 infection through the inhibitory effect of viral M^pro^ activity at a post-entry stage.

### Prophylactic administration of S-217622 confers protection against infection in hamsters

**Fig. 2.**
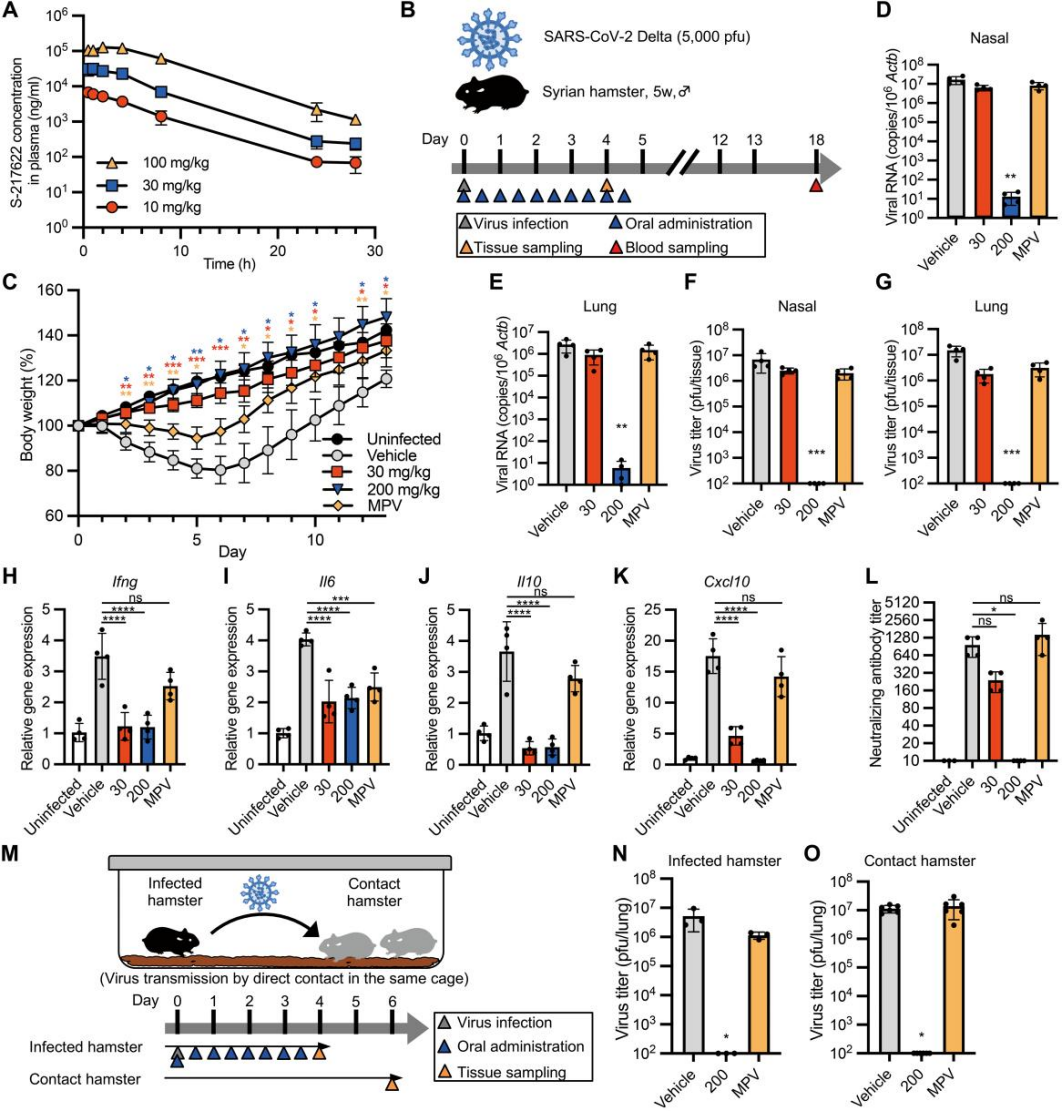
Prophylactic treatment of S-217622 controls viral burden and disease in hamsters inoculated with SARS-CoV-2. (**A**)The plasma concentration profile of S-217622 after a single oral administration in hamsters is shown (n = 3 for each treatment). Plasma samples were harvested at the indicated time points and analyzed by LC-MS/MS. (**B**) Schematic of the experimental design for prophylactic treatment in a hamster model. Hamsters were intranasally inoculated with 5,000 pfu of SARS-CoV-2 Delta variant. For prophylactic treatment, the hamsters were treated with oral administration of S-217622 or vehicle (0 mg/kg) twice daily (b.i.d.) from the time of infection (0 hpi) to 4 dpi. Molnupiravir (MPV) was used as a comparator drug. A group of hamsters (n = 4 for each treatment) was euthanized at 4 dpi for tissue collection. Another subset of hamsters (n = 4 for each treatment; n = 3 for uninfected) was monitored for 13 days for body weight change and then euthanized at 18 dpi for serum collection. (**C**) Body weight changes in uninfected hamsters (n = 3) and SARS-CoV-2-infected hamsters treated with S-217622 (30 mg/kg and 200 mg/kg), vehicle, or MPV (200 mg/kg) is shown (n = 4 for each group). (**D** and **E**) Viral RNA concentrations were measured in nasal turbinates (**D**) and lungs (**E**) isolated from hamsters at 4 dpi. Each group of hamsters was treated with vehicle (red), 30 mg/kg (blue), and 200 mg/kg (orange) of S-217622 or MPV (green) (n = 4 for each group). Relative viral RNA abundance in lungs as compared with lungs from vehicle-treated hamsters were examined. Data were normalized to β-actin. (**F** and **G**) Virus titers were measured in nasal turbinates (**F**) and lungs (**G**) isolated from hamsters at 4 dpi as determined by plaque assay. Each group of hamsters was treated with vehicle (red), 30 mg/kg (blue) and 200 mg/kg (orange) of S-217622, or MPV (green) (n = 4 for each group). (**H** to **K**) Cytokine gene expression was measured in lungs isolated from hamsters at 4 dpi (n = 4 for each group). Relative gene expression of *Ifng* (**H**), *Il6* (**I**), *Il10* (**J**), and *Cxcl10* (**K**) in the lungs was compared to lungs from uninfected hamsters. Data were normalized to *Actb*. (**L**) Neutralizing antibody titers were measured in hamster serum at 18 dpi. (**M**) A schematic of the experimental design for the virus transmission experiment is shown. One hamster per cage was inoculated with 5,000 pfu of SARS-CoV-2 Delta variant (infected hamster). Two naïve hamsters (contact hamsters) were co-housed with the infected hamster. Only the infected hamsters were prophylactically treated with S-217622 or MPV from the time of infection (0 hpi) (b.i.d.) Each treatment group consists of three infected hamsters and six contact hamsters in three cages. (**N** and **O**) Virus titers were measured in lungs isolated from the infected hamsters at 4 dpi (**N**) and isolated from the contact hamsters at 6 days after co-housing (**O**). Each group of the infected hamsters was treated with vehicle (red), 200 mg/kg (blue), or MPV (green). The values shown are mean ± SD. ns, not significant; *p < 0.05, **p < 0.01, ***p < 0.001, ****p < 0.0001 by two-way ANOVA with Dunnett’s test (C), Kruskal-Wallis test with Dunn’s test (D to G, L, N, O) or one-way ANOVA with Tukey’s test (H to K).

We investigated the in vivo antiviral activity of S-217622 using Syrian hamsters as an animal model for COVID-19 (*21*, *22*). The pharmacokinetic parameters in plasma revealed the excellent bioavailability of S-217622 (table S1). After oral administration of S-217622, its concentration in plasma reached the maximum at 0.5 to 2.67 hours after doses of 10, 30, and 100 mg/kg in hamsters, and then declined with half-life (t_1/2,z_) values of 3.43 to 4.46 hours (Fig. 2A and table S1). The maximum concentration (C_max_) and area under the curve (AUC) values were increased more than the dose ratio at 10 to 100 mg/kg. Based on the dose-dependency in hamsters, we set two different dose concentrations (30 mg/kg and 200 mg/kg) for our in vivo experiments.

Hamsters were inoculated with SARS-CoV-2 Delta, a highly pathogenic variant (*13*), followed by oral administration of antivirals twice a day (b.i.d.) from 0 hours post infection (hpi) (Fig. 2B). Control hamsters (0 mg/kg) were orally administered with 0.5% (w/v) methyl cellulose 400 as vehicle only. We employed molnupiravir (MPV, 200 mg/kg) as a comparator drug, which has been reported to reduce the viral load of SARS-CoV-2 in hamsters (*5*, *23*). Control hamsters lost more than 15% of body weight at 4 to 7 days post-infection (dpi), whereas hamsters treated with MPV showed only 5% body weight loss until 6 dpi (Fig. 2C). Hamsters treated with S-217622 continuously gained weight through the experimental period. Viral RNA loads and titers in nasal turbinates and lungs of hamsters treated with antivirals were lower than that of the vehicle-treated group (Fig. 2D to G). Treatment with S-217622 (200 mg/kg) resulted in a more than 10^5^-fold decrease in viral RNA load, and the titers were under the detection limit in hamsters at 4 dpi (Fig. 2F and G). We also compared the antiviral activity of S-217622 and nirmatrelvir using hamsters. In contrast to in vitro assays, S-217622 showed higher antiviral activity than nirmatrelvir in hamsters (fig. S7), presumably due to the pharmacokinetic properties in hamsters (table S1) (*24*).

SARS-CoV-2 infection induced host inflammatory responses in hamsters, whereas treatment with S-217622 decreased the expression of inflammatory cytokines (Fig. 2H to K). Hosts develop specific antibody from 10 to 14 dpi with SARS-CoV-2 (*25*). No seroconversion was observed in hamsters treated with the 200 mg/kg dose of S-217622 (Fig. 2L). These results suggest that S-217622 has antiviral activity in vivo and that prophylactic administration protects hamsters from SARS-CoV-2 infection.

We examined whether prophylactic administration of antivirals to SARS-CoV-2-infected hamsters prevented viral transmission to susceptible hamsters (contact hamster) by co-housing susceptible hamsters with an infected hamster in the same cage (Fig. 2M). Susceptible hamsters continued to be exposed to virus shed from SARS-CoV-2-infected hamsters up to 4 dpi. At 4 dpi, the infected hamsters were euthanized to determine the viral titer in the lungs (Fig. 2N). At 6 days after co-housing, high viral load was detected in the lungs of the hamsters co-housed with infected hamsters treated with vehicle or MPV (Fig. 2O). However, the contact hamsters co-housed with hamsters receiving S-217622 had no detectable viral titers in the lungs. The results suggested that administration of S-217622 can inhibit viral spread among co-housed hamsters, highlighting the high degree of in vivo antiviral efficacy of S-217622.

**Fig. 3.**
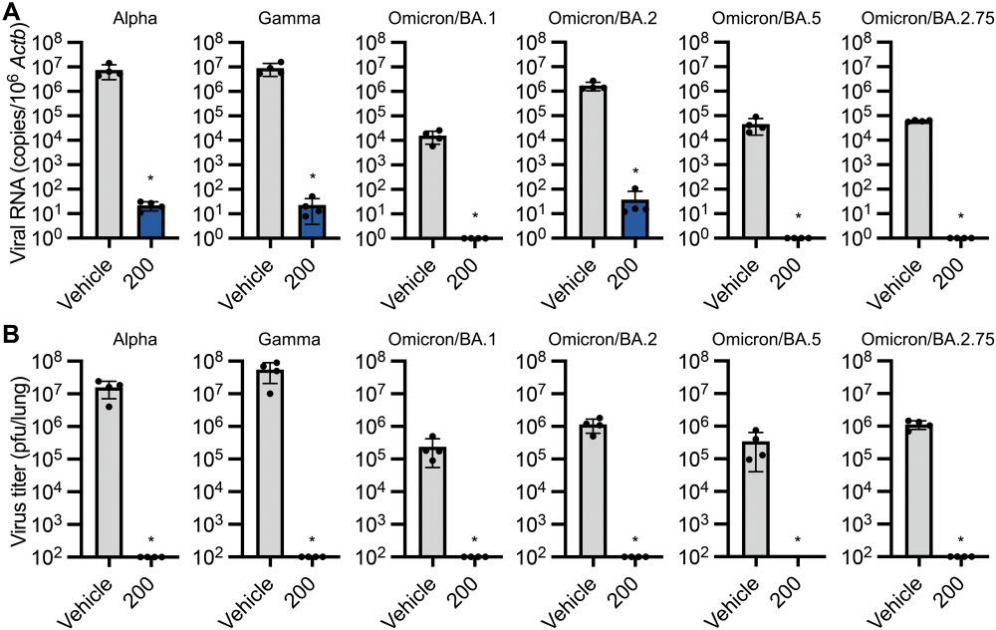
S-217622 retains antiviral activity against SARS-CoV-2 VOCs in vivo. Hamsters were intranasally inoculated with 5,000 pfu of SARS-CoV-2 Alpha, Gamma, and Omicron variants. The hamsters were treated with oral administration of S-217622 (200 mg/kg) or vehicle (0 mg/kg) twice a day from the time of inoculation (0 hpi) to 3 dpi (n = 4 for each group). Lung tissues were harvested at 4 dpi. **(A)** Relative viral RNA abundance in the lungs as compared with lungs from vehicle-treated hamsters were examined. Data were normalized to *Actb*. (**B**) Virus titers in the lungs were measured by plaque assay. The values shown are mean ± SD. *p < 0.05 by two-tailed Mann-Whitney test.

We also examined in vivo antiviral activity of S-217622 against other VOCs: Alpha (lineage B.1.1.7), Gamma (lineage P.1), and Omicron (lineages BA.1, BA.2, BA.5, and BA.2.75), in hamsters (Fig. 3A and B). Hamsters were susceptible to these VOCs and the titers of Omicron in the lungs at 4 dpi were lower than those of other VOCs, consistent with previous reports (*15*, *26*). Oral administration of S-217622 decreased the RNA concentrations and viral loads of all examined VOCs in hamster lungs, indicating S-217622 has antiviral activity against VOCs in vivo.

### Therapeutic administration of S-217622 controls SARS-CoV-2 infection in hamsters

**Fig. 4.**
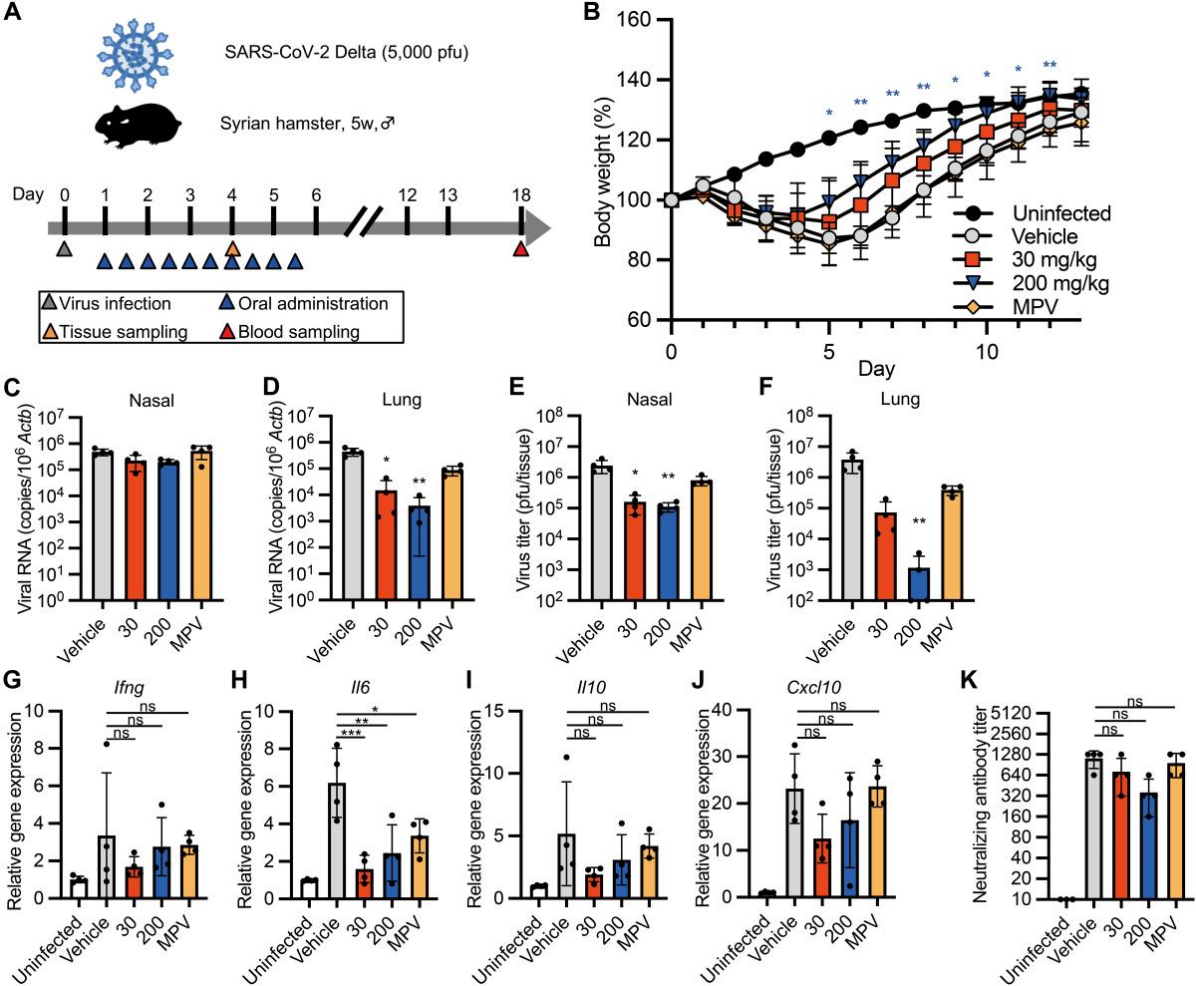
Therapeutic treatment with S-217622 decreased the viral load of SARS-CoV-2 and reduces disease severity in hamsters. (**A**) The experimental design for therapeutic treatment in a hamster model is shown. Hamsters were intranasally inoculated with 5,000 pfu of SARS-CoV-2 Delta variant. For therapeutic treatment, the hamsters were treated with oral administration of S-217622 or vehicle twice daily from 24 hpi to 5 dpi. MPV was used as a comparator drug. A group of hamsters (n = 4 for each treatment) was euthanized at 4 dpi for tissue collection. Another subset of hamsters (n = 4 for each treatment; n = 3 for uninfected) was monitored for 13 dpi for body weight change and then euthanized at 18 dpi for serum collection. (**B**) Body weight change is shown for uninfected hamsters (n = 3) and SARS-CoV-2-infected hamsters treated with S-217622 (30 mg/kg and 200 mg/kg), vehicle, or MPV (200 mg/kg) (n = 4 for each group). (**C** and **D**) Viral RNA concentrations were measured in nasal turbinates (**C**) and lungs (**D**) isolated from hamsters at 4 dpi. Each group of hamsters was treated with vehicle (red), 30 mg/kg (blue) and 200 mg/kg (orange) of S-217622, or MPV (green) from 24 hpi (n = 4 for each group). Relative viral RNA concentrations in lungs were compared with concentrations in lungs from vehicle-treated hamsters. Data were normalized to *Actb*. (**E** and **F**) Virus titers were measured in nasal turbinates (**E**) and lungs (**F**) isolated from hamsters after 4 dpi with SARS-CoV-2 by plaque assay. Each group of hamsters was treated with vehicle (red), 30 mg/kg (blue) and 200 mg/kg (orange) of S-217622, or MPV (green) from 24 hpi (n = 4 for each group). (**G** to **J**) Cytokine gene expression profiles in lungs from hamsters at 4 dpi with SARS-CoV-2 (n = 4 for each group). Relative gene expression of *Ifng* (**G**), *Il6* (**H**), *Il10* (**I**), and *Cxcl10* (**J**) in the lungs was compared with lungs from uninfected hamsters. Data were normalized to *Actb*. (**K**) Neutralizing antibody titers were measured in hamster serum at 18 dpi. The values shown are mean ± SD. ns, not significant; *p < 0.05, **p < 0.01, ***p < 0.001 by two-way ANOVA with Dunnett’s test (B), Kruskal-Wallis test with Dunn’s test (C to F, K) or one-way ANOVA with Tukey’s test (G to J).

SARS-CoV-2 replicates rapidly in hamsters intranasally inoculated with SARS-CoV-2, and the viral load in the upper respiratory tract reaches its peak at 1 to 2 dpi (*13*, *27*). To evaluate the therapeutic efficacy of S-217622, we treated hamsters with S-217622 starting at 24 hpi (delayed treatment protocol) (Fig. 4A). The vehicle and MPV groups showed similar body weight curves and lost more than 10% of body weight at 5 to 6 dpi (Fig. 4B). In contrast, the peak of body weight loss in hamsters receiving S-217622 was 7% (30 mg/kg) and 5% (200 mg/kg) and body weight recovery was accelerated by the administration of S-217622. Administration of S-217622 or MPV had a limited effect on viral loads in nasal turbinates, but these antivirals decreased the viral loads in the lungs at 4 dpi (Fig. 4C to F). Administration of S-217622 (200 mg/kg) achieved a greater than 10^3^-fold decrease of virus titer in the lung of hamsters at 4 dpi (Fig. 4F). Gene expression analysis by quantitative real-time polymerase chain reaction (qRT-PCR) revealed that administration of S-217622 decreased the expression of *Il6*, but the overall reduction of inflammation was limited (Fig. 4G to J). In contrast to prophylactic administration, seroconversion was observed in all groups of hamsters infected with SARS-CoV-2 (Fig. 4K).We amplified and sequenced the *nsp5* gene from progeny viruses in the lungs from S-217622-treated hamsters at 4 dpi. The nucleotide sequences of progeny viruses were identical with original Delta variant (GISAID: EPI_ISL_2158617). These results suggest that administration of S-217622 decreases the viral load in the lungs and facilitates recovery from infection even with delayed treatment.

### S-217622 reduces evidence of lung damage in SARS-CoV-2-infected hamsters

**Fig. 5.**
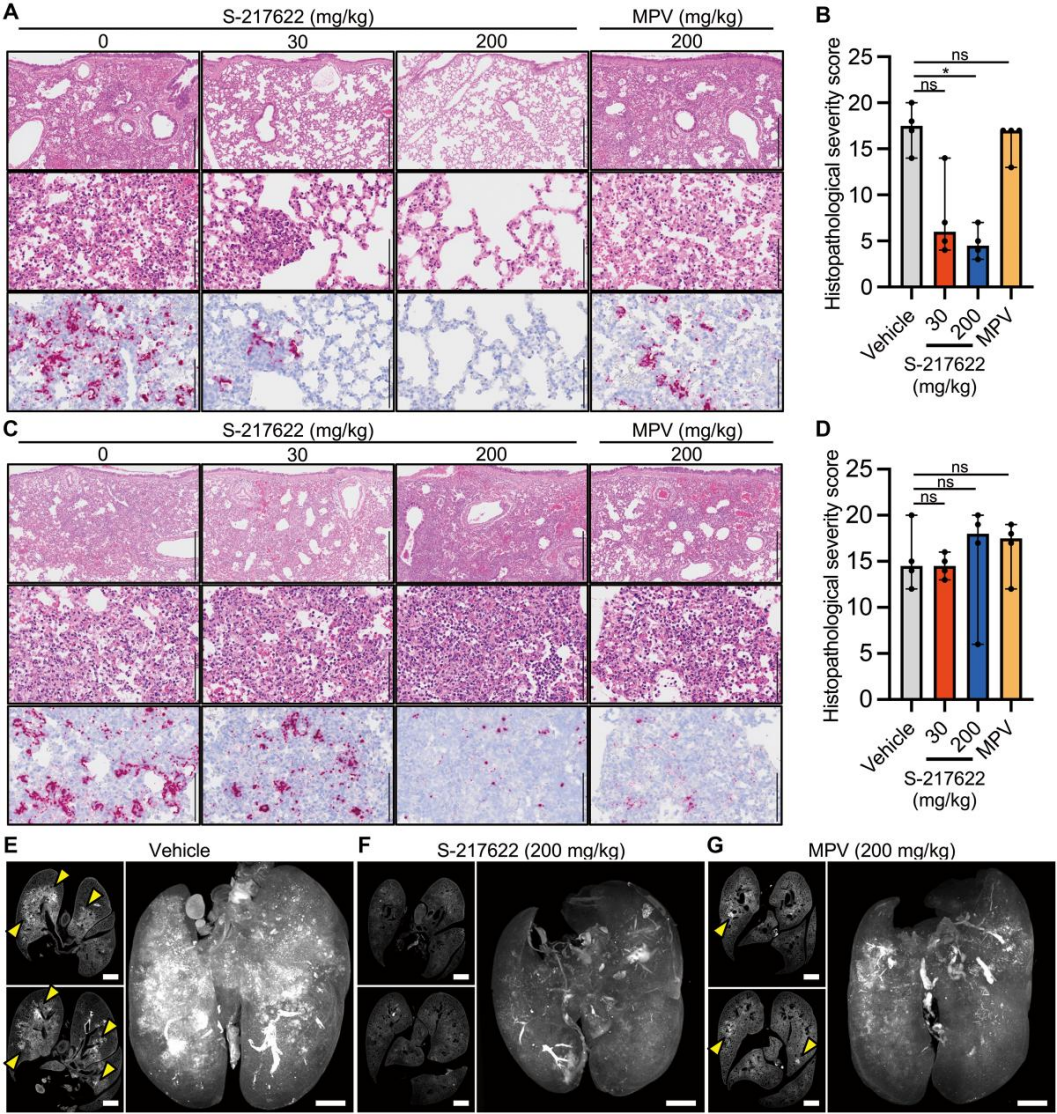
Histopathological analysis of the lungs isolated from SARS-CoV-2-infected, antiviral-treated hamsters. Hamsters were infected with 5,000 pfu of SARS-CoV-2 Delta variant and euthanized at 4 dpi for histopathological examination. S-217622 (30 mg/kg or 200 mg/kg), molnupiravir (200 mg/kg), or vehicle control was administered from 0 to 3 dpi in the prophylactic setting (**A** and **B**) or 1 to 3 dpi in the therapeutic setting (**C** to **G**) twice a day following the schedules shown in Fig. 2B and Fig. 3A, respectively. (**A** and **C**) Representative histopathological images are shown for lung sections obtained from the animals treated with the indicated antivirals (n = 4 for each group). Upper and middle panels, hematoxylin and eosin (H&E) staining. Lower panels, ISH targeting the *n* gene of SARS-CoV-2. Scale bars in upper panels, 500 μm. Scale bars in middle and lower panels, 100 μm. (**B** and **D**) Histopathological severity score of pneumonia was calculated based on the percentage of alveolitis area in a given section. Data are shown as the median score ± 95% confidential interval with each dot representing the score of each animal (n = 4). ns, not significant; *p < 0.05 by Kruskal-Wallis test with Dunn’s test. (**E** to **G**) Shown are cross-sectional images of lungs and 3D image reconstruction of whole lungs isolated from hamsters at 4 dpi. Infected hamsters were treated with vehicle (**E**), S-217622 (**F**), or MPV (**G**). The whole lung tissues were stained with anti-SARS-CoV-2 spike protein antibody and scanned by light sheet microscopy. Arrowheads indicate foci of SARS-CoV-2-positive alveoli. Scale bars, 2 mm.

In the prophylactic experiments (Fig. 2B), histopathological findings of viral pneumonia were prominent among the vehicle group and the MPV-administered hamsters (Fig. 5A). In contrast, S-217622 attenuated the lung pathology in a dose-dependent manner (Fig. 5A), and the histopathological severity score of the S-217622-administered (200 mg/kg) group was lower than the vehicle control group (Fig. 5B). Viral RNA was decreased following both MPV and S-217622 (30 mg/kg) administration, and was undetectable following S-217622 (200 mg/kg) administration, as measured by in situ hybridization (ISH) (Fig. 5A), consistent with the results of the qRT-PCR assay (Fig. 2E).

In the therapeutic experiments, histopathological examination of the lungs of vehicle control hamsters revealed massive infiltration of inflammatory cells, including neutrophils and lymphocytes, into alveoli and the alveolar walls, which was accompanied with alveolar hemorrhage (Fig. 5C). These findings were also detected in the drug-administered hamsters (Fig. 5C), and neither MPV or S-217622 could attenuate the histopathological severity of viral pneumonia by therapeutic treatment (Fig. 5D). Viral RNA was readily detected by ISH across extended areas of the lungs from the vehicle group (Fig. 5C). Positive signals for viral RNA were also detected in both of S-217622- and MPV-administered animals (Fig. 5C). The histopathological severity of viral pneumonia was similar between the mock-treated and antiviral-treated groups (Fig. 5D).

The viral antigen distributions in the lungs of the therapeutic administered groups were investigated using whole-imaging approaches with light sheet microscopy. Whole lung tissues were fixed, bleached, and stained with anti-SARS-CoV-2 antibody and then exposed to clearing solution for scanning. Slice images showed bronchial and peribronchial distribution of viral antigen in vehicle-treated hamsters (Fig. 5E). Reconstructed 3D images revealed that virus infection spread throughout the lungs of the vehicle-treated controls (Fig. 5E and Movie S1). Consistent with the results of ISH, the signal distribution of viral antigen was limited in the lungs of hamsters treated with S-217622 (Fig. 5F and Movie S2). Administration of MPV also decreased the viral distribution (Fig. 5G and Movie S3). Taken together, these findings suggested that SARS-CoV-2 replication was inhibited by both prophylactic and therapeutic administration of S-217622, and that the progression of lung pathology due to viral pneumonia was also suppressed by prophylactic administration of S-217622.

## DISCUSSION

In this study, we characterized the antiviral activity of S-217622, a newly identified SARS-CoV-2 M^pro^ inhibitor. Our in vitro experiments revealed that S-217622 exhibits remarkable antiviral potency against all VOCs. Current vaccines and monoclonal antibody medications target the viral spike protein, which has accumulated amino acid variations as new VOCs and VOIs have arisen (*16*, *17*). In contrast, the viral M^pro^ is less divergent, leading to comparable susceptibilities of VOCs to S-217622. It has been shown that S-217622 exerts antiviral activity against other coronaviruses: SARS-CoV (effective concentration; EC_50_ = 0.21 μM), MERS-CoV (EC_50_ = 1.4 μM), and HCoV-OC43 (EC_90_ = 0.074 μM) (*9*). Although host factors involved in virus proliferation could be targets for antivirals, viral proteins are specific and favorable targets for antiviral development (*28*). Overall, SARS-CoV-2 M^pro^ is a prospective target and the inhibitor S-217622 is a broadly reactive antiviral against SARS-CoV-2 variants.

Prophylactic administration of S-217622 prominently decreased viral load in both nasal turbinates and lung tissues. Hamsters receiving high doses of S-217622 developed no detectable neutralizing antibodies at 18 days after inoculation. This result indicates that administration of S-217622 confers sterile protection against SARS-CoV-2 in the recipient animal and suggests the potential of S-217622 as a preventive medication for high-risk individuals who have close contact with patients with COVID-19, in addition to the potential treatment of those with active disease. In animal experiments, prophylactic administration at pre-infection or infection stages is a highly sensitive experimental method to detect the efficacy of antivirals (*29*, *30*). Our experiments showed that S-217622 has an anti-SARS-CoV-2 effect even in a post-exposure treatment environment, highlighting the antiviral activity of S-217622.

MPV and nirmatrelvir have been approved as oral antiviral medications for clinical use; however, concerns remain regarding the efficacy and potential risks. The results of a recent clinical trial showed that MPV treatment decreased the risk of hospitalization for COVID-19 by 30% (*31*). Our experiments also showed that therapeutic treatment with MPV had a limited effect on viral load and body weight decrease in hamsters. It also has been reported that MPV has mutagenesis not only for viral RNA but also for host DNA in cell-based assays (*32*). Nirmatrelvir is another M^pro^ inhibitor and is reported to have an excellent in vivo efficacy, with an 88% reduction in the risk of hospitalization or death (*33*). A previous study, along with our data, shows that nirmatrelvir is highly sensitive to the multidrug transporter, P-gp, and requires a P-gp inhibitor to exhibit activity in Vero cells (*12*). For clinical use, nirmatrelvir requires co-administration with a CYP3A4 inhibitor (ritonavir) as a pharmacokinetic booster to slow the metabolism of nirmatrelvir. Because the ritonavir booster also affects the metabolism of other medications, clinicians need to consider the potential other drug-drug interactions during treatments (*34*). In contrast, S-217622 showed expected bioavailability and concentration without any pharmacokinetic booster for humans in a phase 1 trial (*35*). Consequently, S-217622 has different biological properties from the preceding medications and is expected to be an alternative candidate for COVID-19 therapy.

We note some limitations of our study. First, therapeutic treatment with S-217622 was insufficient to control pneumonia in SARS-CoV-2-infected hamsters, although they regained lost body weight earlier compared to vehicle controls. We assume that the initial virus proliferation stimulated host immunity and subsequent inflammation (*36*, *37*), and combination with antiviral and anti-inflammatory medication may lead to better results and outcomes (*38*). Second, this study was conducted using hamsters as a COVID-19 model and the efficacy in human patients cannot be inferred. However, S-217622 is currently under evaluation in a phase 3 clinical trial. Third, the development of a resistant viral clone against S-217622 and its virological properties will need to be investigated in future studies. In summary, our study has demonstrated the remarkable antiviral activity of S-217622 through in vitro and in vivo experiments. This scientific evidence will be essential when considering the application of S-217622 as a medication for COVID-19.

## MATERIALS AND METHODS

### Study design

This study was designed to characterize the antiviral activity of S-217622 using several variants of SARS-CoV-2. All experiments with infectious SARS-CoV-2 were performed in biosafety level 3 laboratory at the International Institute for Zoonosis Control, Hokkaido University. The animal experiments with virus infection were performed in accordance with the National University Corporation, Hokkaido University Regulations on Animal Experimentation. The protocol was reviewed and approved by the Institutional Animal Care and Use Committee of Hokkaido University (approval no. 20–0060). The animal experiments for pharmacokinetics analysis were approved by the Director of the institute after reviewing the protocol by the Institutional Animal Care and Use Committee in SHIONOGI & CO., Ltd. (approval no. S21187C-0001). Experiments were done at least twice. All experiments with biological replicates were indicated in figure legends.

### Cells

Calu-3 cells from the American Type Culture Collection (ATCC, HTB-55) were maintained in Eagle’s Minimum Essential Medium (MEM) containing 10% fetal bovine serum (FBS). Vero-TMPRSS2 cells [Vero E6 cells (ATCC, CRL-1586) stably expressing human TMPRSS2] and Vero-human angiotensin converting enzyme 2 (hACE2)-TMPRSS2 [Vero E6 cells stably expressing human ACE2 and human TMPRSS2] were established as described previously (*39*, *40*) and maintained in Dulbecco’s Modified Eagle’s Medium (DMEM) containing 10% FBS. 293 T cells from the RIKEN BioResource Center (BRC, RCB2202) were maintained in high glucose DMEM containing 10% FBS. 293 T-ACE2-TMPRSS2 cells stably expressing human TMPRSS2 and ACE2 were established by a lentiviral vector transduction system as previously described (*41*) and maintained in high glucose DMEM containing 10% FBS. Differentiated HBE cells were obtained as MucilAir-bronchial (Epithelix, EP01) and maintained under an air-liquid interphase culture condition with MucilAir culture medium (Epithelix) according to manufacturers’ instruction.

### Viruses

SARS-CoV-2 VOC Alpha (strain QK002, lineage B.1.1.7, GISAID: EPI_ISL_768526), Beta (strain TY8–612, lineage B.1.351, GISAID: EPI_ISL_1123289), Gamma (strain TY7–501, lineage P.1, GISAID: EPI_ISL_833366), Delta (strain TY11–927, lineage B.1.617.2, GISAID: EPI_ISL_2158617), Omicron (strain TY38–873, lineage BA.1, GISAID: EPI_ISL_7418017; strain TY40–385, lineage BA.2, GISAID: EPI_ISL_9595859; strain TY41–702, lineage BA.5, GISAID: EPI_ISL_13241867; strain TY41–716, lineage BA.2.75, GISAID: EPI_ISL_13969765) were provided from National Institute of Infectious Diseases, Japan. The working viral stocks were prepared by a passage on Vero-TMPRSS2 or Vero-hACE2-TMPRSS2 cells. The titers of the prepared virus stocks were determined as plaque forming unit per ml (pfu/ml) by a plaque assay.

### Compounds

S-217622 (also known as ensitrelvir, fumaric acid co-crystal form) and nirmatrelvir were synthesized by Shionogi & Co., Ltd. (*9*). MPV and remdesivir were obtained from MedChemExpress. NHC was obtained from Angene. REGN10933 and REGN10987 were obtained from Cell Sciences. CP-100356 was obtained from Santa Cruz Biotechnology.

### Plaque assays

Monolayers of Vero-TMPRSS2 were inoculated with serial dilutions of either virus stock or clarified tissue homogenates for 1 hour at 37°C. The cells were then overlaid with DMEM containing 2% FBS, 0.5% Bacto Agar (Becton Dickinson) and 25 μg/ml gentamicin (Wako). At 3 dpi with omicron and 2 dpi with other variants, cells were fixed with 3.7% formaldehyde in phosphate-buffered saline (PBS) and stained with 1% crystal violet. For titration of BA.2, BA.5, and BA.2.75, Vero-hACE2-TMPRSS2 cells were used instead of Vero-TMPRSS2.

### Viral RNA and host mRNA measurements

Vero-TMPRSS2 and Calu-3 cells were inoculated with SARS-CoV-2 at multiplicities of infection (MOIs) of 0.01 or 0.1, respectively. After 1 hour of incubation, the cells were washed three times with PBS and fed with fresh culture medium containing 2% FBS and S-217622. The culture supernatants were harvested at 24 hpi from Vero-TMPRSS2 cells and 72 hpi from Calu-3 cells. The MOIs and harvest time points were optimized to achieve similar infection rates in different cell lines. Viral RNA in the supernatant was extracted with High Pure Viral RNA kit (Roche) and quantified by qRT-PCR with Thunderbird Probe One-step qRT-PCR Kit (Toyobo). The viral copy number was estimated by the standard curve method. Probe and primers targeting SARS-CoV-2 *N* gene were previously described as N2 set (*42*).

To assess the relationship between P-gp and SARS-CoV-2 main protease inhibitors, Vero-TMPRSS2 cells were infected with SARS-CoV-2 Delta variant at an MOI of 0.01 and treated with S-217622 at 100 nM or nirmatrelvir at 300 nM for 24 hours in the absence or presence of P-gp inhibitor, CP-100356 at 1 μM. Total RNA was extracted from cells with Purelink RNA Mini kit (Invitrogen) and subjected to qRT-PCR assay as described above.

For measurement of cytokine gene expression and viral RNA in hamsters, total RNA was extracted from a part of whole tissue homogenates with a combination of TRIzol LS (Invitrogen) and Direct-zol RNA MiniPrep kit (Zymo Research). For relative quantification of viral RNA and host mRNAs, RNA samples were analyzed by qRT-PCR with Thunderbird Probe One-step qRT-PCR Kit. Target RNA concentrations were normalized to hamster β-actin and calculated by the relative standard curve method. Primers and probes for SARS-CoV-2 *N* gene were noted above. Primers and probes for *Actb*, *Ifng*, *Il6*, *Il10*, and *Cxcl10* (*43*) were previously described.

### Cytopathic effect-based cell viability assays

Antiviral compounds and antibodies were serially diluted 2-fold increments by culture medium containing 2% FBS and plated on 96-well microplates. The diluted compounds in the plates were mixed with SARS-CoV-2 and cell suspensions. The diluted antibodies were initially incubated with SARS-CoV-2 for 30 minutes and then added to the cell suspension. Cells in the plates were cultured for 2 to 3 days, and then exposed to MTT (3-[4,5-dimethyl-2-thiazolyl]-2,5-diphenyl-2H-tetrazolium bromide) (Nacalai Tesque). Cell viability was determined by measurement of absorbance at 560 nm and 690 nm. EC_50_ values were defined in GraphPad Prism version 8.4.3 (GraphPad Software) with a variable slope (four parameters). Non-treated cells were used as a control for 100% inhibition.

### Immunofluorescence staining

Vero-TMPRSS2 and Calu-3 cells were inoculated with SARS-CoV-2 at MOIs of 0.1 and 10, respectively. After a 1 hour incubation, cells were fed with fresh culture medium containing 2% FBS and S-217622. Vero-TMPRSS2 cells at 24 hpi and Calu-3 cells at 72 hpi were fixed with 3.7% formaldehyde in PBS. The MOIs and harvest time points were optimized to achieve similar infection rates in different cell lines. Cells were then permeabilized with 0.5% Triton X-100 in PBS for 5 minutes and stained with anti-SARS-CoV-2 nucleocapsid rabbit monoclonal antibody (1:1000, GTX635679, GeneTex) in 25% Block Ace (KAC) in PBS for 1 hour. Alexa Fluor 488-conjugated anti-rabbit IgG antibody (1:1000, Invitrogen; Thermo Fisher Scientific) was used as the secondary antibody. Nuclei were stained with Hoechst 33342 (Invitrogen). Fluorescent images were captured using IX73 fluorescence microscope (Olympus).

### Evaluation of antiviral efficacy S-217622 in human bronchial epithelial cells

The apical area of human bronchial epithelial cells were washed with culture medium and then inoculated with 5,000 pfu of SARS-CoV-2. After 30 minutes of incubation, the apical area was washed with PBS and the basal medium was replaced with fresh culture medium supplemented with S-217622. At 24, 48, and 72 hpi, 100 μl of culture medium was added at the apical area and harvested for virus titration after a 20 minute incubation. At 72 hpi, the concentration of LDH in the basal culture medium was quantified by LDH-Glo Cytotoxicity Assay kit (Promega).

### Time of addition assay

Calu-3 cells were inoculated with SARS-CoV-2 Delta variant at an MOI of 3 for 2 hours in the presence or absence of inhibitors [S-217622 (1 μM), camostat (50 μM, Wako) or anti-SARS-CoV-2 spike protein-specific monoclonal antibody (1 μg/ml, GTX635792, GeneTex)]. After washing three times with PBS, cell were cultured for 5 hours with or without inhibitors. Total RNA was extracted from the cells using Purelink RNA Mini kit (Invitrogen) and subjected to measurement of intracellular viral RNA concentrations by qRT-PCR. Target RNA concentrations were normalized to expression of the gene encoding human β-actin (Hs99999903_m1, Applied Biosystems; Thermo Fisher Scientific) and calculated by the ΔΔCt method. Primers and probes for SARS-CoV-2 *N* gene were as noted above.

### Pharmacokinetics studies

Five-week old male Syrian hamsters (Japan SLC) were orally administered 10, 30, or 100 mg/kg of S-217622 under the non-fasting conditions (n = 3 per dose). Dosing vehicles were 0.5% (w/v) methyl cellulose 400. Multiple plasma samples (0.1 mL) were collected over time from hamsters and plasma samples were stored in a freezer until analysis. Plasma concentrations of S-217622 were determined by liquid chromatography-tandem mass spectrometry (LC-MS/MS) system following protein precipitation with acetonitrile (MeCN). The LC-MS/MS system, equipped with a positive electrospray ionization (ESI) surface, consisted of an API5000 mass spectrometer (AB SCIEX) and a Nexera liquid chromatograph (Shimadzu Corporation). The multiple reaction monitoring (MRM) mode was selected, and the precursor ion (m/z 532.347) and product ion (m/z 145.042) were monitored. The cone voltage and collision energy were set at 90 V and 55 V, respectively. A YMC-Triart C18 (3 μm, 2.1 mm I.D. × 50 mm) column (YMC Co., Ltd) was used and the column temperature was maintained at 40°C for chromatographic separation of analytes. The mobile phases were 0.1% (v/v) formic acid in distilled water (mobile phase A) and MeCN (mobile phase B). The flow rate was 0.75 mL/minute. The gradient condition was 30–65–95-95-30 (% of mobile phase B concentration) / 0 0.9–0.91-1.1-1.11-1.5 (minute). Pharmacokinetic parameters of S 217622 in plasma were calculated by WinNonlin (Ver. 8.3, Certara, L.P.) based on a non-compartment analysis with uniform weighting.

### Virus infection and treatment of hamsters

Five-week old male Syrian hamsters (Japan SLC) were intranasally inoculated with 5,000 pfu of SARS-CoV-2 in 200 μl of PBS under anesthesia with isoflurane inhalation. S-217622 was suspended in 0.5% (w/v) methyl cellulose 400. MPV was suspended in 10% polyethylene glycol 400 with 2.5% Cremophor RH40. Nirmatrelvir was suspended in corn oil with 2% t-Butyl Methyl Ether. Prophylactic and therapeutic treatments were started immediately after virus inoculation (0 hpi) or 24 hpi, respectively. Hamsters were orally treated twice daily with antivirals under anesthesia with isoflurane inhalation. Vehicle control hamsters were administered 0.5% (w/v) methyl cellulose 400. For virus titration and gene expression assays, nasal turbinates and lungs were collected from a subset of hamsters (n = 4 for each treatment) at 4 dpi and homogenized in 1 ml or 5 ml of PBS with TissueRuptor (Qiagen), respectively. A part of the homogenate was subjected to plaque assays for virus titration. Total RNA was extracted from the homogenate with Direct-zol RNA MiniPrep kit and analyzed by qRT-PCR as mentioned above. Another subset of hamsters (n = 4 for each treatment; n = 3 for uninfected) were monitored for up to 13 dpi for body weight change. Body weights of animals were monitored daily. To determine the titers of neutralizing antibody in hamsters, serum samples were collected from hamsters at 18 dpi and heat-inactivated at 56°C for 30 minutes. Virus neutralization assays were performed following the protocol as previously described (*44*). The neutralization titers were defined as the reciprocal of the highest serum dilution that completely inhibited the cytopathic effect in Vero-TMPRSS2 cells.

For virus transmission between animals, one hamster per cage was inoculated with 5,000 pfu of SARS-CoV-2 Delta variant (infected hamster) and co-housed with two naïve hamsters (contact hamsters) in the same cage. Only infected hamsters were orally treated twice daily with antivirals from the time of infection (0 hpi). Each treatment group consisted of three infected hamsters and six contact hamsters in three cages. Lung tissues were sampled from infected hamsters at 4 dpi and contact hamsters after 6 days of co-housing with the infected hamster. Viral loads were analyzed by virus titration and qRT-PCR as described above.

### Histopathological examination

Lung tissues were harvested at 4 dpi. Tissues were fixed in 3.7% formaldehyde in PBS and embedded in paraffin. To detect viral RNA in the paraffin sections, ISH was carried out using an RNA scope 2.5 HD Red Detection kit (Advanced Cell Diagnostics) with antisense probe targeting the *N* gene of SARS-CoV-2 (Advanced Cell Diagnostics) as previously described (*15*). Histopathological severity score of pneumonia was determined in a non-blinded manner based on the percentage of alveolar inflammation in a given area of a pulmonary section collected from each animal in each group using the following scoring system: 0, no pathological change; 1, affected area ≤ 10%; 2, affected area < 50%, >10%; 3, affected area ≥ 50%; an additional point was added when pulmonary edema or alveolar hemorrhage was observed (*15*). The total score for the five lobes was calculated for each animal.

### Light sheet microscopy

Samples for whole imaging analysis were prepared following the method of iDISCO as previously described with minor modifications (*45*). Lung tissues collected at 4 dpi were fixed in 3.7% formaldehyde in PBS. The lungs were dehydrated by serial incubations in PBS, 50% methanol in PBS, 80% methanol in PBS, and 100% methanol for 90 minutes each. Tissue bleaching was performed in a 9:1 mixture of 100% methanol and 30% H_2_O_2_ solution for overnight at 4°C. The lungs were rehydrated by serial incubations in 100% methanol twice, 80% methanol in PBS, 50% methanol in PBS, and PBS for 60 minutes each. Tissue blocking was performed in PBSBT (20% Block Ace, 0.5% Triton X-100, 0.009% NaN_3_ in PBS) for 24 hours. Tissues were then stained with anti-SARS-CoV-2 spike protein antibody (1:2000, GTX635792, GeneTex) in PBSBT with 0.1% saponin for 4 days, followed by washing five times for 12 minutes each in PBS with 0.5% Triton X-100. Tissues were further incubated for 4 days with secondary antibody staining solution consisting of Alexa Fluor Plus 647-conjugated anti-rabbit IgG (1:1000, A32795, Invitrogen) diluted in PBSBT with 0.1% saponin and filtrated though a 0.45 μm syringe filter. After washing five times for 12 minutes each in PBS with 0.5% Triton X-100, the immunostained tissues were dehydrated by serial incubations in 50% methanol in PBS, 80% methanol in PBS, and 100% methanol twice for 12 hours each. Samples were then treated with dichloromethane for 40 minutes for removal of lipids, and then dibenzyl ether overnight. All incubation processes were conducted on an orbital shaker or a rotator. Fluorescence images were acquired by UltraMicroscope Blaze (Miltenyi Biotec) according to the manufacturers’ instructions. Image data was converted and processed into 3D reconstruction by Imaris software (version 9.8.0, Oxford Instruments).

### Statistical analysis

Raw, individual-level data are presented in data file S1. Statistical significance was determined by two-tailed Mann-Whitney test (Fig. 3A and B), one-way analysis of variance (ANOVA) with Dunnett’s test (Fig. 1D, 1E, 1H, 1I; fig. S5B and S6), one-way ANOVA with Tukey’s test (Fig. 2H to K, and 4G to J), two-way ANOVA with Dunnett’s test (Fig. 2C  and 4B), or Kruskal-Wallis test with Dunn’s multiple comparisons test (Fig. 1G, 2D to G, 2L, 2N, 2O, 4C to F, 4K, 5B, 5D; fig. S2 and S7). All statistical tests were carried out using Prism version 8.4.3 (GraphPad software).

## Supplementary Materials

### Other Supplementary Material for this manuscript includes the following:

Data file S1

MDAR Reproducibility Checklist

## Notes

View/request a protocol for this paper from *Bio-protocol*.
