# Supplementary material for: S-217622, a SARS-CoV-2 main protease inhibitor, decreases viral load and ameliorates COVID-19 severity in hamsters
Source: Sci Transl Med. 2022 Nov 3:eabq4064. doi: 10.1126/scitranslmed.abq4064 (PMC9765455; doi:10.1126/scitranslmed.abq4064)
Supplement: Supplementary file 1 — Materials and Methods Figs. S1 to S7 Table S1 [file scitranslmed.abq4064_sm.pdf]

Science Translational Medicine
AAAS

Supplementary Materials for

S-217622, a SARS-CoV-2 main protease inhibitor, decreases viral load and ameliorates COVID-19 severity in hamsters

Michihito Sasaki *et al.*

Corresponding author: Michihito Sasaki, m-sasaki@czc.hokudai.ac.jp; Hirofumi Sawa, h-sawa@czc.hokudai.ac.jp

Sci. Transl. Med. **0**, eabq4064 (2022)
DOI: 10.1126/scitranslmed.abq4064

The PDF file includes:

Materials and Methods
Figs. S1 to S7
Table S1

Other Supplementary Material for this manuscript includes the following:

Movies S1 to S3
Data file S1
MDAR Reproducibility Checklist

Supplementary Materials and Methods

Compound cytotoxicity assay

Cells were treated with S-217622 serially diluted in 2-fold increments by culture medium containing 2% fetal bovine serum (FBS). After a 3 day culture, cell toxicity was evaluated by measurement of ATP in live cells with CellTiter-Glo 2.0 reagent (Promega) or lactate dehydrogenase (LDH) released from dead cells in the basal medium with LDH-Glo Cytotoxicity Assay kit (Promega). Luminescence was detected on a luminometer (GloMax-Multi detection system, Promega).

Biosensor assay for viral protease activity

The DNA fragments encoding NSP3 or NSP4, NSP5, N-terminal NSP6 (NSP4/5/6N) of SARS-CoV-2 WK-521 strain (lineage A) were cloned into pCMV-derivative pCXSN vector to generate pSARS-CoV-2 papain-like protease (PL^{pro}) and pSARS-CoV-2 main protease (M^{pro}), respectively. The amino acid sequence AVLQS (for the cleavage by M^{pro}) or RLKGG (for the cleavage by PL^{pro}) were replaced into pGloSensor-30F vector backbone (CS182101, Promega) to generate firefly luciferase-based biosensor expressing plasmid pGS-AVLQS or pGS-RLKGG, respectively. 293T cells on 96-well plate were co-transfected with a set of pGS-AVLQS and pSARS-CoV-2 M^{pro} or a set of pGS-RLKGG and pSARS-CoV-2 PL^{pro} using TranIT-LT1 (Mirus Bio) in the presence of serial diluted S-217622. At 24 hours post-transfection, the luminescence signals of biosensors and renilla luciferase (an internal control reporter) from pGS vectors were measured by Dual-Glo Luciferase Assay System (Promega). The ratio (firefly luciferase:renilla luciferase) was calculated to normalize for the influence of transfection efficiency. Non-treated cells were used as a control for 100% biosensor activation.

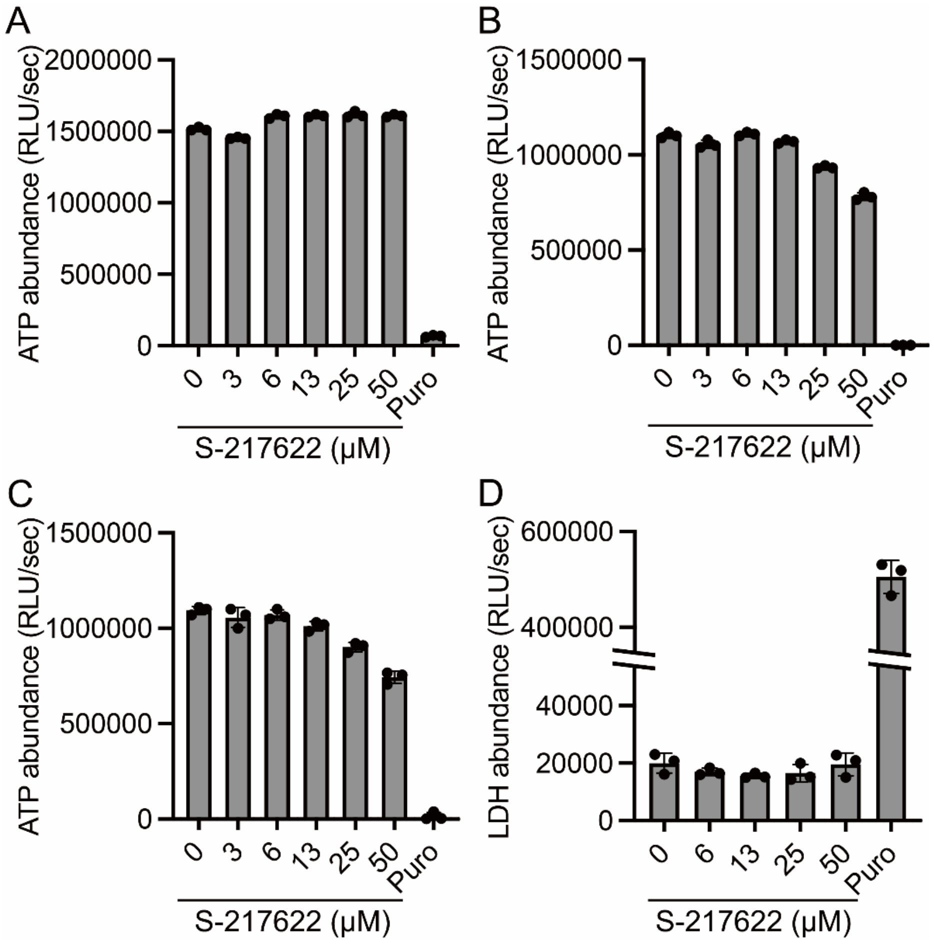

Fig. S1. Cytotoxicity of S-217622.

(**A to D**) Calu-3 cells (A), Vero-TMPRSS2 cells (B), Vero-TMPRSS2 cells with CP-100356 at 1 μM (C), and primary human bronchial epithelial cells (D) were treated with S-217622 for 3 days. Cell toxicity was evaluated by measurement of ATP in live cells (A to C) or LDH released from dead cells in the basal medium (D). Puromycin (puro) was used as a control compound to mediate cell toxicity. The values shown are mean ± SD of triplicate samples. RLU, relative light units.

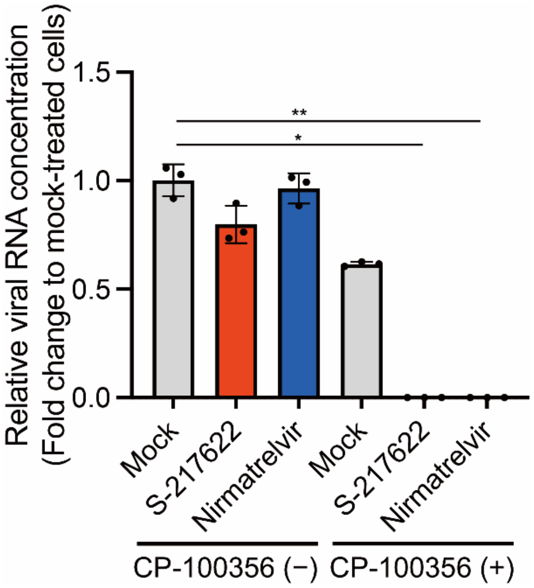

Fig. S2. Antiviral activity of SARS-CoV-2 main protease inhibitors in the presence of a P-glycoprotein (P-gp) inhibitor.

Vero-TMPRSS2 cells were infected with SARS-CoV-2 Delta variant at a multiplicity of infection (MOI) of 0.01 and treated with S-217622 at 100 nM or nirmatrelvir at 300 nM for 24 hours in the absence or presence of the P-gp inhibitor, CP-100356 at 1 μM. The inhibitory effect of each treatment was evaluated by measurement of intracellular viral RNA concentration at 24 hours post infection (hpi). The values shown are mean ± SD of triplicate samples. *$p<0.05$, **$p<0.01$ by Kruskal-Wallis test with Dunn's test.

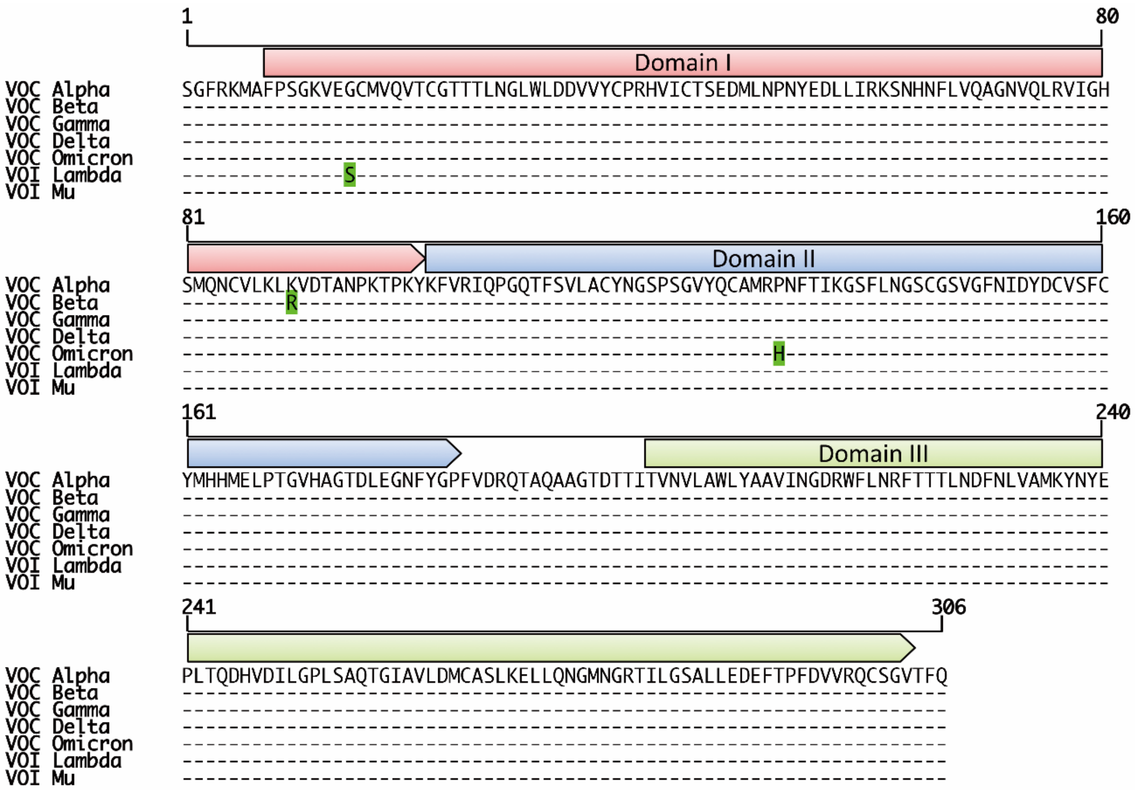

Fig. S3. Multiple amino acid sequence alignments of M^{pro} from SARS-CoV-2 variants of concern (VOCs) and variants of interest (VOIs).

Multiple sequence alignment based on the entire length of M^{pro} from the VOCs Alpha (lineage B.1.1.7, GISAID: EPI_ISL_768526), Beta (lineage B.1.351, GISAID: EPI_ISL_1123289), Gamma (lineage P.1, GISAID: EPI_ISL_877769), Delta (lineage B.1.617.2, GISAID: EPI_ISL_2158617), and Omicron (lineage BA.2.75, GISAID: EPI_ISL_14011362), as well as the VOIs Lambda (lineage C.37, GISAID: EPI_ISL_4204973), and Mu (lineage B.1.612, GISAID: EPI_ISL_4470503). Dashes (-) represent amino acids identical to M^{pro} of Alpha. Amino acids different from the consensus are colored in green.

A

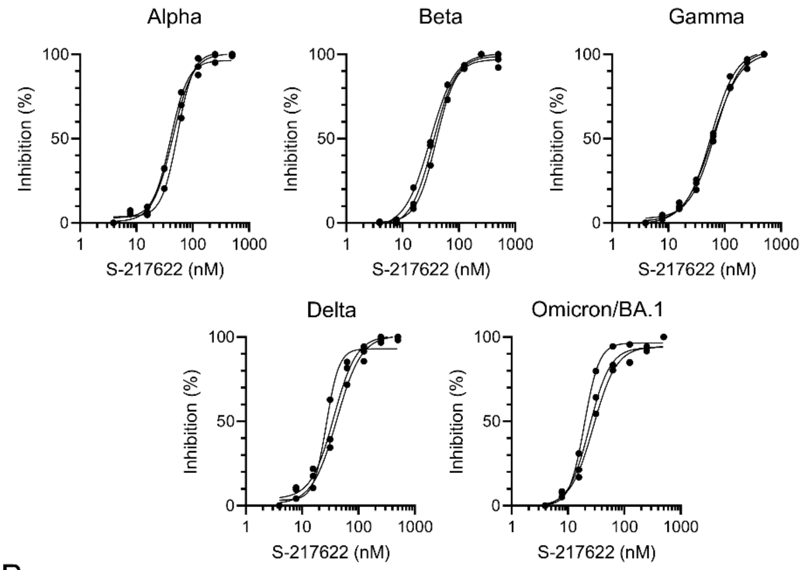

B

	EC50					CC50
	Alpha	Beta	Gamma	Delta	Omicron /BA.1	
**S-217622 (nM)**	46.0±5.5	35.1±3.5	61.7±3.9	34.8±5.9	23.9±3.6	>50000
**REGN10933 (ng/ml)**	16.9±2.9	>1000	>1000	6.3±1.8	>1000	>1000
**REGN10987 (ng/ml)**	3.7±0.3	3.4±0.8	8.4±1.8	8.6±3.9	>1000	>1000

Fig. S4. Antiviral potency of S-217622 against SARS-CoV-2 VOCs.
Cytopathic effect (CPE) in 293T-hACE2-TMPRSS2 cells induced by SARS-CoV-2 infection was measured at 3 dpi by 3-[4,5-dimethyl-2-thiazolyl]-2,5-diphenyl-2H-tetrazolium bromide (MTT) assay. (**A**) The inhibitory effect of S-217622 on SARS-CoV-2 VOCs was examined. Uninfected cells were used as a control for 100% inhibition. (**B**) The 50% effective concentration (EC_{50}) and cytotoxic concentration (CC_{50}) of S-217622 and anti-SARS-CoV-2 neutralizing monoclonal antibodies (REGN10933 and REGN10987) are shown.

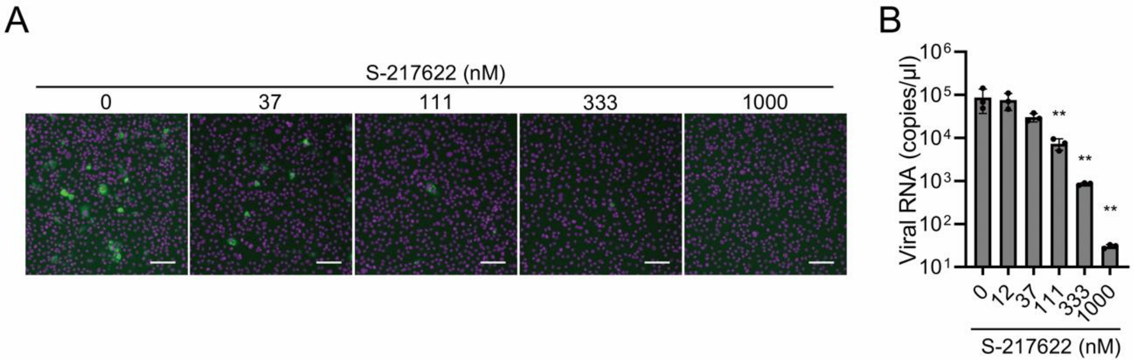

Fig. S5. Susceptibility of the SARS-CoV-2 Omicron variant to treatment with S-217622.
(**A**) Immunofluorescence staining is shown of Vero-TMPRSS2 cells infected with the SARS-CoV-2 Omicron variant BA.1. Cells were inoculated with the SARS-CoV-2 Omicron variant at an MOI of 0.1 and then cultured in the presence of S-217622 for 24 hours. Cells were stained with anti-SARS-CoV-2 nucleocapsid antibody (green) and Hoechst 33342 (magenta). Scale bars, 100 µm. (**B**) Viral RNA concentrations were measured in the culture supernatant of Vero-TMPRSS2 at 24 hpi with the SARS-CoV-2 Omicron variant at an MOI of 0.01. The values shown are mean ± SD of triplicate samples. **p<0.01 by one-way ANOVA with Dunnet's test.

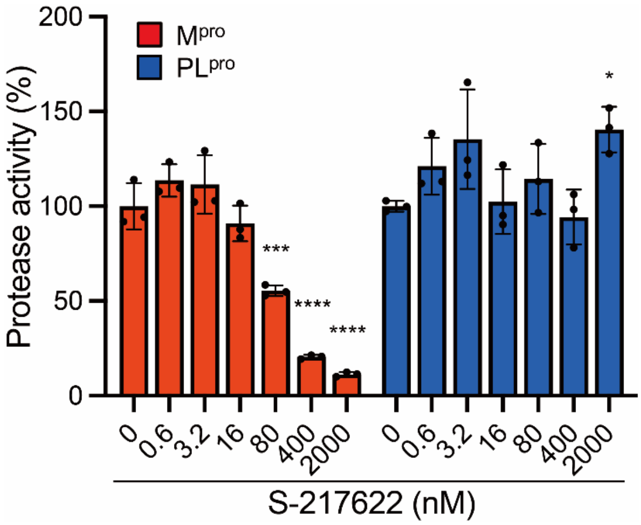

Fig. S6. Luciferase-based biosensor assay for SARS-CoV-2 protease activity.

293T cells were co-transfected with expression plasmids of SARS-CoV-2 M^{pro} and a biosensor for SARS-CoV-2 M^{pro} and maintained in the presence of S-217622. The luminescence signal from the biosensor was measured as the protease activity at 24 hours post transfection. 293T cells expressing SARS-CoV-2 PL^{pro} and another biosensor for PL^{pro} were used as a control for the target specificity of S-217622. The values shown are mean ± SD of triplicate samples. *$p<0.05$, ***$p<0.001$, ****$p<0.0001$ by one-way ANOVA with Dunnett's test.

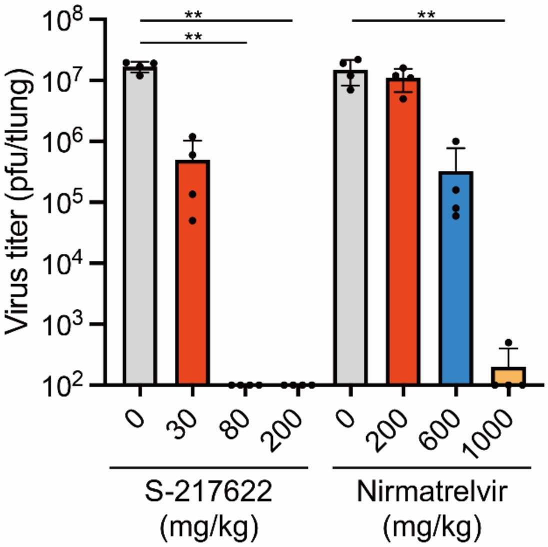

Fig. S7. In vivo antiviral efficacy of SARS-CoV-2 main protease inhibitors.

Hamsters were intranasally inoculated with 5,000 plaque-forming units (pfu) of the SARS-CoV-2 Delta variant. The hamsters were treated with oral administration of S-217622 or nirmatrelvir twice daily (b.i.d.) from the time of infection (0 hpi) for prophylactic treatment (n=4 for each treatment). Virus titers in lungs from hamsters at 4 dpi were determined by plaque assay. The values shown are mean ± SD. **p<0.01 by Kruskal-Wallis test with Dunn's test.

Table S1. Pharmacokinetic parameters of S-217622 in plasma after a single oral administration of S-217622 in hamsters.

	S-217622 in plasma		
	10 mg/kg	30 mg/kg	100 mg/kg
C_{max} (μM)	12.7±2.2	61.6±18.7	254±24
T_{max} (h)	0.5±0.0	1.17±0.76	2.67±1.15
$t_{1/2,z}$ (h)[a]	4.46±1.03	3.96±0.74	3.43±0.13
$AUC_{0\text{-}28h}$ (μM·h)	78.3±11.0	415±52	2480±350
$AUC_{0\text{-}inf}$ (μM·h)	79.3±10.5	417±51	2500±350

a: $t_{1/2,\ 8\text{-}28hr}$

C_{max}, maximum concentration; T_{max}, time to peak drug concentration; $t_{1/2}$, half-life; AUC, area under the curve.

Movie S1. Reconstructed 3D image of viral antigen distribution in the lung of a vehicle-treated hamster at 4 dpi. The hamster was inoculated with SARS-CoV-2 Delta variant and treated with 0.5% methyl cellulose solution (vehicle for S-217622) from 1 dpi to 3 dpi. Related to Fig. 5E.

Movie S2. Reconstructed 3D image of viral antigen distribution in the lung of a S-217622-treated hamster at 4 dpi. The hamster was inoculated with SARS-CoV-2 Delta variant and treated with S-217622 (200 mg/kg) from 1 dpi to 3 dpi. Related to Fig. 5F.

Movie S3. Reconstructed 3D image of viral antigen distribution in the lung of a MPV-treated hamster at 4 dpi. The hamster was inoculated with SARS-CoV-2 Delta variant and treated with MPV (200 mg/kg) from 1 dpi to 3 dpi. Related to Fig. 5G.

Data file S1. Raw, individual-level data for all experiments.
